# Intelligent Resource Allocation for V2V Communication with Spectrum–Energy Efficiency Maximization

**DOI:** 10.3390/s23156796

**Published:** 2023-07-29

**Authors:** Chunning Xu, Shumo Wang, Ping Song, Ke Li, Tiecheng Song

**Affiliations:** 1 School of Architecture, Urban Planning & Design Institute, Southest University, Nanjing 210096, China; xuchunning@seu.edu.cn; 2 School of Information Science and Engineering, Southeast University, Nanjing 210096, China; 220210900@seu.edu.cn (S.W.); 101009964@seu.edu.cn (P.S.); 3 Smart City College, Beijing Union University, Beijing 100101, China; like@buu.edu.cn; 4 Shenzhen Research Institute, Southeast University, Shenzhen 518057, China

**Keywords:** vehicular networking, resource allocation, 5G network slicing, multi-agent deep Q learning

## Abstract

Aiming to address the limitations of traditional resource allocation algorithms in the Internet of Vehicles (IoV), whereby they cannot meet the stringent demands for ultra-low latency and high reliability in vehicle-to-vehicle (V2V) communication, this paper proposes a wireless resource allocation algorithm for V2V communication based on the multi-agent deep Q-network (MDQN). The system model utilizes 5G network slicing technology as its fundamental feature and maximizes the weighted spectrum–energy efficiency (SEE) while satisfying reliability and latency constraints. In this approach, each V2V link is treated as an agent, and the state space, action, and reward function of MDQN are specifically designed. Through centralized training, the neural network parameters of MDQN are determined, and the optimal resource allocation strategy is achieved through distributed execution. Simulation results demonstrate the effectiveness of the proposed scheme in significantly improving the SEE of the network while maintaining a certain success rate for V2V link load transmission.

## 1. Introduction

Among the fundamental technologies in intelligent transportation systems (ITS), the Internet of Vehicles (IoV) serves as a platform for information transmission, facilitating the creation of a vast network for the exchange and sharing of information among vehicle-to-everything (V2X) entities. Through real-time sensing and collaboration among various functional entities, such as people, vehicles, roads, and clouds, IoV has become a crucial tool, reducing traffic congestion, enhancing operational efficiency, and promoting safe and eco-friendly travel in modern cities [1,2,3].

With the advent of the 5G era, the number of IoV users and services is expected to increase substantially. In-vehicle mobile terminal communication devices will be required to not only process massive volumes of service data but also ensure the quality requirements of differentiated and diverse services. Networked technologies are essential to realize L3 level conditional autonomous driving and L4 advanced autonomous driving [4], compensating for the limited sensing capability of local sensors and enabling the fusion decision making of global sensing information in complex environments.

L3 and L4 level autonomous driving systems impose significant demands for quality of service (QoS). However, several challenges exist in ensuring QoS through resource allocation. On one hand, autonomous driving relies on immediate and reliable traffic information, requiring 99.999% reliability and ultra-low latency of less than 5 ms end-to-end [5]. Hence, it is crucial to allocate limited network resources appropriately to guarantee QoS for users. On the other hand, the proliferation of IoV applications and the densification of communication node deployments have led to a substantial increase in the variety and volume of services in next-generation traffic information networks. The exponential growth of data further exacerbates resource constraints. Wireless resource allocation methods for IoV can be categorized into three types: those based on traditional convex optimization theory, game theory, and machine learning-based approaches.

Resource allocation optimization is a challenging problem, commonly formulated as a mixed-integer nonlinear programming model, known for its nonconvexity and NP-hardness [6]. Previous studies [7,8,9] have primarily addressed resource allocation using traditional convex optimization principles. In [7], a mixed-integer nonlinear programming problem is formulated to maximize the sum ergodic capacity of vehicle-to-infrastructure (V2I) links while ensuring a specified probability of delayed outages in vehicle-to-vehicle (V2V) links. The problem is decomposed into two subproblems: power allocation and spectrum allocation. For dynamic resource allocation [8], a two-stage algorithm named DRA and DRA-Pre is introduced, which utilizes a multi-valued discrete particle swarm optimization technique to solve the resource allocation problem in the first stage, while dividing the precoding design problem into rate maximization and total power consumption minimization subproblems in the second stage. In [9], whale optimization algorithms are applied to address resource allocation problems in wireless networks. This involves power allocation strategies to achieve a balance between energy and spectral efficiency, power allocation to maximize throughput, and mobile edge computing shunting. Alternatively, certain studies [10,11,12] treat the resource allocation problem as a game and utilize game theory techniques to solve it.

One study [10] proposes an innovative auction-matching-based spectrum allocation scheme that considers interference constraints and user satisfaction. This approach effectively improves the spectrum efficiency for users, addressing the limitations of traditional mechanisms. In a different study [11], the resource allocation problem is modeled as a multi-user game, and the existence of Nash equilibrium is proven through a potential game. To optimize the computational and communication resources and maximize the system utility, a multi-user offloading algorithm based on better response is proposed. In yet another study [12], a resource allocation scheme is designed for unmanned aerial vehicle (UAV)-assisted vehicle communication scenarios. The study introduces a two-stage resource allocation algorithm based on Stackelberg game principles. In the first stage, a clustering algorithm matches users with different blocks of spectrum resources. Subsequently, in the second stage, the Stackelberg game is employed to solve the power optimization problem for each cluster, achieving the dual objectives of maximizing the sum rate of V2I users while ensuring the reliability of V2V users.

Deep learning has emerged as a powerful data-driven approach addressing resource allocation challenges by learning efficient data representations with multiple levels of abstraction from unstructured sources [13]. In [14], a deep learning-based damped 3D messaging algorithm is proposed, considering tradeoffs between energy efficiency and spectral efficiency as the optimization objective. This algorithm takes into account quality of service, power consumption, and data rate constraints. In [15], a binarized neural network is utilized for resource allocation. The primary objective of this scheme is to maximize the classification accuracy at the server while adhering to a total transmit power constraint. Another study [16] introduces a distributed resource allocation mechanism where agents, such as V2V links or vehicles, can make decisions without waiting for global state information. Each agent efficiently learns to satisfy strict delay constraints on V2V links while minimizing interference with V2I communication. Considering the bandwidth and low latency requirements in vehicular communication applications, one study [17] proposes a framework and optimization scheme for the slicing pf networks based on device-to-device communication. The slicing resource allocation problem is modeled as a Markov decision process, and a deep reinforcement learning (RL) algorithm is employed to solve the problem, leading to improved resource utilization, slice satisfaction, and throughput gain. In the integration of communication mode selection and resource allocation, one study [18] formulates a Markov decision process problem and solves it using a deep deterministic policy gradient algorithm. This approach effectively improves the long-term energy efficiency. For joint computation offloading and resource allocation decisions, another study [19] proposes two distinct methods: a value iteration-based RL method and a double deep Q-network (DDQN)-based method. These methods aim to optimize resource allocation decisions while considering computation offloading. Additionally, in [20], a novel fuzzy-logic-assisted Q-learning model (FAQ) is proposed, leveraging the advantages of the centralized allocation mode. The FAQ model aims to maximize network throughput while minimizing interference caused by concurrent transmissions during resource allocation.

Additionally, 5G New Radio V2X (NR V2X) [21] is considered a promising technology after LTE V2X, proposing new goals for air interface selection, interface enhancement, and quality management to support advanced V2X applications with varying levels of latency, reliability, and throughput requirements. The application of network slicing technology [22] provides a novel idea for telematics resource allocation. V2I and V2V communication aim to facilitate information exchange, promoting enhanced mobile broadband and ultra-reliable low-latency communication for widespread usage. Activities such as cloud access, video streaming, and in-vehicle social networking entail large data exchanges, necessitating frequent access to wireless access points and core web servers, requiring high-speed communication links for efficient data transmission. Similarly, safety-critical information, such as collaboration-aware content, dispersed environmental notification information, and autonomous driving data in vehicle networks, demand exceptionally stringent ultra-low latency and high reliability. Consequently, V2V communication needs to offer communication services with improved spectrum and energy efficiency and higher communication rates and meet more stringent reliability and latency requirements by accessing ultra-reliable and low-latency communications (URLLC) slices.

Traditional resource allocation methods can no longer meet the delay and reliability demands of V2V communication. In recent years, with the development of artificial intelligence, researchers have found reinforcement learning [23] to be effective for decision making in situations with uncertainty conditions. It provides a robust and principled approach to making a series of decisions in dynamically changing environments, making it a considerable approach to address specific highly dynamic problems in vehicular networking.

Hence, studying the V2V resource allocation problem with the ultra-low latency and high reliability constraints of V2V communication holds significant research significance. The main work and innovations of this paper are as follows.

- We explore the application of mixed spectrum access for V2V links and V2I links under the 5G New Radio V2X standard and network slicing technology. To maximize the spectrum–energy efficiency (SEE) of the network, we incorporate the reliable transmission and delay constraints of URLLC slicing into the optimization problem.
- We use the multi-agent deep Q-network (MDQN) to handle the resource allocation problem by setting up state, action, and reward functions rationally.
- Simulation results demonstrate that the proposed algorithm improves the SEE of the network while guaranteeing the success rate of V2V link load transmission. When compared to three other resource allocation schemes, the MDQN algorithm allows for distributed resource allocation in response to environmental variations, promoting collaboration among V2V links to achieve global optimization.

## 2. System Model

Based on 5G NR V2X, in order to improve the network transmission rate and enhance the spectrum utilization at the same time, a hybrid spectrum access technology is proposed to be used for transmission, i.e., the PC5 interface and the Uu interface share spectrum resources. Vehicles can be divided into two types: cellular users (CUEs), who communicate with the base station for V2I and access the vehicular network through the Uu interface to request high-speed communication services from the base station, and V2V users (VUEs), who communicate with adjacent vehicles for side-chain communication and access the vehicular network through the PC5 interface to achieve low latency and high reliability. To cater to diverse vehicle requirements, we establish two logical slices within a shared infrastructure: the eMBB slice and the URLLC slice. Within the 5G network, vehicles have the option to access either the eMBB slice or the URLLC slice. The eMBB slice is designed for regular internet access and remote server connectivity, involving substantial data exchange. It serves CUEs that require frequent and reliable internet access. On the other hand, the URLLC slice is intended for the transmission of safety-critical information and is primarily utilized by VUEs.

**Figure 1:**
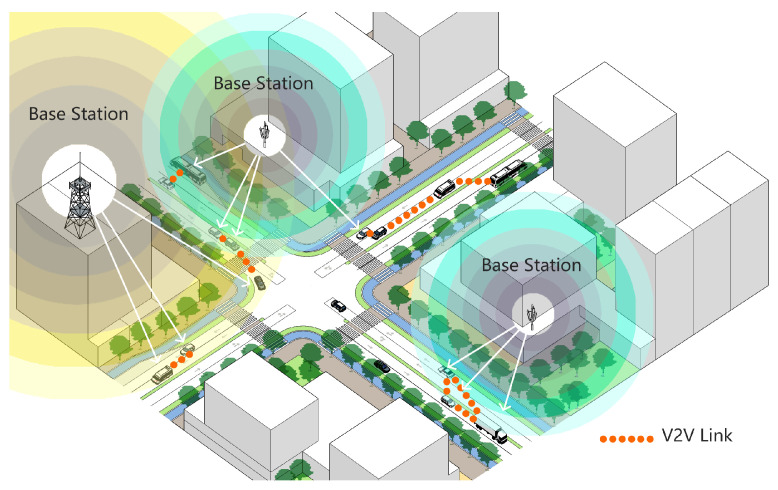
Schematic diagram of 5G telematics V2X communication.

Since V2V and V2I communications utilize different slices, the resource allocation for V2V links must be independent of the resource allocation for V2I links. Figure 1 illustrates a V2X communications scenario based on the 5G network, where *K* pairs of V2V links are present. For V2V links, we assume that there is an authorized bandwidth of *B*, which is evenly divided into *M* subchannels. Denote the subchannel set and the V2V link set as M={1,2,...,M} and K={1,2...,K}, respectively. At the same time, the channel transmission in the model uses Orthogonal Frequency Division Multiplexing (OFDM) technology, and the subchannels are orthogonal to each other without interference, but the same subchannel can be shared by more than one user and interference will occur between the VUEs sharing the same subchannel, thus affecting the channel capacity.

The signal to interference plus noise ratio (SINR) of the *k*th V2V link at the *m*th subchannel can be expressed as

(1) $$\gamma_{{}^{k}}^{v}\left[m\right]=\frac{P_{k}^{v}\left[m\right]g_{k}\left[m\right]}{I_{k}\left[m\right]+\sigma^{2}}.$$

where $g_{k}\left[m\right]$ represents the channel gain of the *k*th V2V link at the *m*th subchannel; it comprises two components: the large-scale fading component and the small-scale component. Additionally, the large-scale fading component includes two factors, namely shadowing and path loss. The channel capacity of the *k*th V2V link on the *m*th subchannel can be expressed as

(2) $$C_{{}^{k}}^{v}\left[m\right]=W\log\left(1+\gamma_{k}^{{}^{v}}\left[m\right]\right).$$

Among them,

(3) $$I_{k}\left[m\right]=\sum_{k^{\prime }\in K,k^{\prime }\neq k}\rho_{k^{\prime }}\left[m\right]P_{k^{\prime }}^{v}\left[m\right]{\tilde{g}}_{k^{\prime },k}\left[m\right],$$

$I_{k}\left[m\right]$ is the total interference power of all V2V links sharing the same subchannel. $P_{k}^{v}\left[m\right]$ denotes the transmit power of the *k*th VUE. $\sigma^{2}$ denotes the noise power, and ${\tilde{g}}_{k^{\prime },k}^{}\left[m\right]$ is the interference gain of the $k^{\prime }$th V2V link to the *k*th V2V link. $\rho_{k}\left[m\right]$ indicates the subchannel allocation indicator, and $\rho_{k}\left[m\right]=1$ indicates that the *k*th V2V link occupies the subchannel *m*; otherwise, $\rho_{k}\left[m\right]=0$. It is specified that each V2V link can select only one subchannel for transmission at the same moment, i.e., $\sum_{m=1}^{M}\rho_{k}^{}\left[m\right]=1,\forall 1\leq k\leq K$.

In addition, the reliability requirement of V2V communication is expressed by the following equation.

(4) $$\sum_{m=1}^{M}\rho_{k}^{}\left[m\right]\gamma_{{}^{k}}^{v}\left[m\right]>\gamma_{TH},\forall 1\leq k\leq K,$$

where $\gamma_{TH}$ is the signal-to-noise ratio threshold for the VUE receiver on the *k*th V2V link.

Assume that the packet arrival process of the *k*th V2V link is independently and identically distributed and obeys a Poisson distribution with an arrival rate of $\lambda_{k}$. $N_{k}\left(x\right)$ denotes the size of the first *n* packets and obeys an exponential distribution with an average packet size of ${\bar{N}}_{k}\left(x\right)$, and $Q_{k}\left(t\right)$ is the number of packets cached by the *k*th V2V link in the time slot *t*. Moreover, $W_{k}\left(x\right)$ is the packet waiting time in the cache to be served, and $\delta_{k}\left(x\right)$ denotes the packet transmission time, so the time delay of the *x*th packet in the V2V user’s *k* cache is

(5) $$D_{k}\left(x\right)=W_{k}\left(x\right)+\delta_{k}\left(x\right).$$

The packet of the *k*th V2V link must be guaranteed to be transmitted within a finite time, $d_{max}$ denotes the maximum tolerable delay in packet transmission, δ is the maximum violation probability, and the delay interruption probability of V2V communication is bounded by

(6) $$P\left\{D_{k}\left(x\right)>d_{\max}\right\}\leq \delta ,\forall k=1,2,\cdots ,K.$$

The constraints on the physical layer metrics of V2V include the spectral efficiency and energy efficiency, defined as the channel capacity that can be obtained per unit frequency and per unit energy consumption. Thus, the spectrum–energy efficiency (SEE) of V2V links can be expressed as

(7) $$\zeta^{{}_{V2V}}=\frac{\sum_{k=1}^{K}\sum_{m=1}^{M}C_{k}^{v}\left[m\right]}{\sum_{m=1}^{M}W\times \left(\sum_{k=1}^{K}\sum_{m=1}^{M}P_{k}^{v}\left[m\right]+KP_{c}\right)},$$

where $P_{c}$ is the circuit power.

The objective of V2V resource allocation is to allocate the V2V link transmission power and subchannels to maximize the SEE of the V2V links while satisfying the delay and reliability constraints. Therefore, the following objective function and constraints can be established.

(8) $$\begin{array}{cc} \; & \max\zeta^{V2V}\\ \;s.t.\; & C1:\sum_{m=1}^{M}\rho_{k}\left[m\right]\gamma_{k}^{v}\left[m\right]>\gamma_{TH},\forall 1\leq k\leq K\\ \; & C2:P\left\{D_{k}\left(x\right)>d_{\max}\right\}\leq \delta ,k=1,2,\ldots ,K\\ \; & C3:\sum_{m=1}^{M}p_{k}^{v}\left[m\right]\leq p_{\max},\forall k=1,2,\ldots ,K\\ \; & C4:\rho_{k}\left[m\right]\in \left\{0,1\right\},\forall m=1,2,\ldots ,M,\forall k=1,2,\ldots ,K\\ \; & C5:\sum_{m=1}^{M}\rho_{k}\left[m\right]=1,\forall k=1,2,\ldots ,K \end{array},$$

where the objective function is to maximize the SEE of the V2V links, constraints C1 and C2 are reliability and delay constraints on the V2V links, constraint C3 states that the transmission power is within the reachable maximum transmission power, and constraints C4 and C5 imply that each V2V link can be assigned to only one subchannel, but the same subchannel can access multiple V2V links.

## 3. Resource Allocation Algorithm

**Figure 2:**
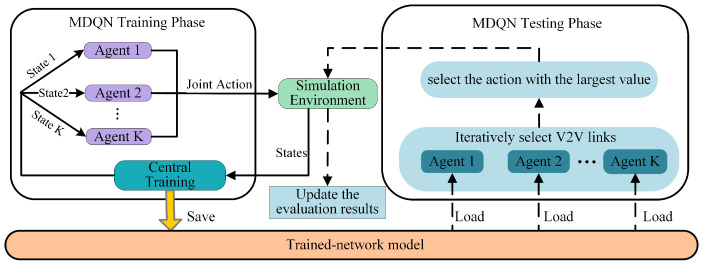
Flowchart of the MDQN algorithm.

Reinforcement learning is a powerful technique that can be used to solve optimization problems. In the field of resource management for V2V communication, the conventional Q-learning approach has been observed to encounter difficulties in achieving convergence of the Q-function due to the limited accessibility of states and infrequent updates of corresponding Q-values. This limitation has led to the development of a more efficient method known as the deep Q-network (DQN), which combines the Q-learning algorithm with deep neural networks. We leverage DQN to train the multiple V2V agents, namely multi-agent DQN (MDQN). Motivated by the literature [24], the MDQN algorithm comprises two distinct phases: the MDQN training phase and the MDQN testing phase, as depicted in Figure 2. During the training phase, each V2V link operates as an independent agent, engaging in interactions with the simulation environment to obtain rewards. In order to ensure consistency in network performance, all agents are assigned the same reward. Moreover, the reward and subsequent state are solely dependent on the current state and the joint actions taken by all agents [25]. This information, which is introduced as follows, encompassing the current state, action, reward, and subsequent state, is then utilized to update the network of each agent. In the MDQN testing phase, each V2V agent receives local observations of the environment and, based on its trained network model, selects an action to execute.

### 3.1. Status, Action, Reward

**Figure 3:**
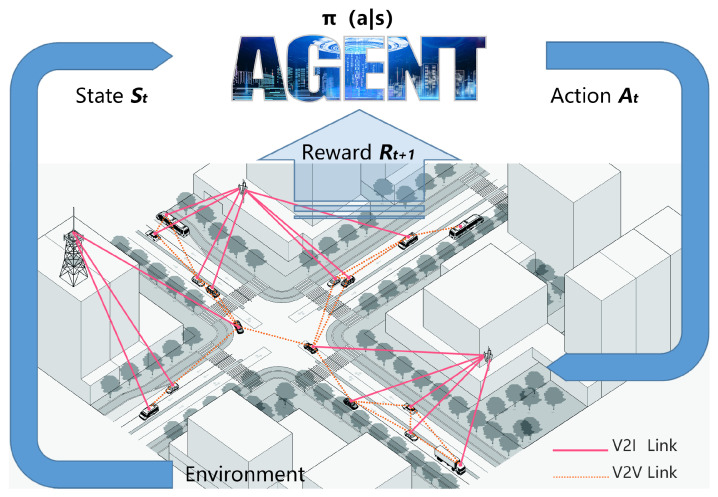
Deep reinforcement learning model for vehicle network resource allocation.

The high mobility of vehicles can result in incomplete access to channel state information for the central controller, which necessitates the use of a distributed resource allocation scheme. This framework considers each V2V link as an agent, with all other V2V links being regarded as the environment. At any given moment *t*, the state of an agent is represented by the information that it senses, denoted as $S_{t}$. Agents interact with the environment by selecting subchannels and transmission power based on their local observations. This interaction results in the agent receiving a reward $r_{t}$ and a new state $s_{t+1}$, as shown in Figure 3.

State information $S_{k}$ includes the local instantaneous channel information of the subchannel *m*, denoted as $G_{k}\left[m\right]$, the remaining V2V payload $B_{k}$, and the remaining time budget $T_{k}$.

(9) $$G_{k}\left[m\right]=\left\{g_{k}\left[m\right],{\left\{{\tilde{g}}_{k^{\prime },k}^{}\left[m\right]\right\}}_{k^{\prime }\neq k}\right\},$$

(10) $$S_{k}=\left\{{\left\{G_{k}\left[m\right]\right\}}_{m\in M},B_{k},T_{k}\right\},$$

where $g_{k}\left[m\right]$ is the channel power gain of the *k*th V2V link, and ${\tilde{g}}_{k^{\prime },k}^{}\left[m\right]$ is the interference of the $k^{\prime }$ th V2V link with the *k*th V2V link.

Action space *A* includes the selection subchannel and the transmit power, and the selection subchannel is denoted by $\rho_{k}\left[m\right]$. $\rho_{k}\left[m\right]=1$ denotes the *k*th V2V link using the *m*th channel. In existing resource allocation schemes, most of the transmission power is set to a continuous reading value, but considering the model training and actual vehicle limitations, the transmission power is set to [23,18,10,5,−100] dBm, and when the V2V link selection transmission power level is −100 dBm, it indicates that the V2V link transmission power level is 0. Thus, the action space dimension is 5×M, and each action corresponds to a particular combination of subchannel and power selection.

The flexibility of the reward function is a key strength of deep reinforcement learning, as it enables the agent to learn and adapt its behavior by receiving feedback in the form of rewards from its environment. This process of learning through trial and error can lead to significant improvements in performance, as the agent becomes better equipped to achieve its objectives.

The objective of V2V resource allocation is to maximize the SEE of the network while ensuring that the V2V link load is transmitted within the maximum tolerable delay and considering the SINR threshold value of the link. Therefore, the design of the reward function needs to consider these three components and the expression is as follows.

(11) rt=∑k=1Krt(k)/K,rt(k)=ζV2V+λ3G(γkv−γd)+λ4G(∑m=1Mρk[m]Ckv[m]−BkBkTkTk),ifBk>0,A1,oherwise.

Among them,

(12) $$G\left(x\right)=\left\{\begin{array}{cc} A_{2}, & ifx>0,\\ x, & otherwise. \end{array}\right.$$

where $A_{1}$ is a fixed large constant, $A_{2}$ is also a constant, and $\lambda_{3}$ and $\lambda_{4}$ are weights, both of which are empirically adjusted hyperparameters.

### 3.2. MDQN Algorithm

The Q-value, denoted by Q(s,a), represents the expected long-term reward for taking an action *a* in a given state *s*. The classical Q-learning approach involves constructing a table of state–action pairs to store the Q-values and selecting actions based on these values to obtain larger gains. The algorithm works by initializing a table of values, setting the initial state, selecting the current state action with the highest reward based on the table, executing the action *a*, and observing the resulting return *r* and next state $s^{\prime }$. The algorithm then updates the Q-value table for each step by calculating Q(s,a) and storing it in the table. The Q-value can be considered as the expectation of the long-term payoff and the update formula can be expressed as

(13) $$Q\left(s,a\right)\leftarrow Q\left(s,a\right)+\alpha (r+\gamma \max_{a^{\prime }}Q\left(s^{\prime },a^{\prime }\right)-Q\left(s,a\right))$$

where α is the learning rate that controls the step size of the updates and γ is the discount factor that determines the importance of future rewards.

DQN leverages the approximation of the optimal Q-values by the network with parameters denoted by θ through the following equation.

(14) $$f\left(s,a,\theta \right)=Q^{*}\left(s,a\right)$$

In training neural networks, DQN proposes two unique mechanisms.

**Figure 4:**
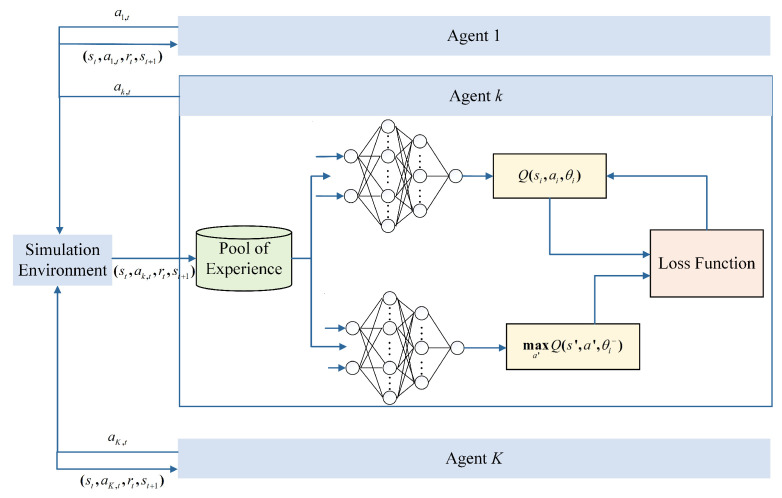
Block diagram of MDQN algorithm based on deep reinforcement learning.

1. Experience replay is a technique whereby the agent stores its experiences in a replay buffer, which is a data structure that contains a collection of transitions $\left(s,a,r,s^{\prime }\right)$. The agent then samples a batch of transitions from the replay buffer and uses them to update its Q-network. This approach leads to a reduction in the correlations between successive updates, allowing for the more efficient use of data and improving the stability.
2. The target network involves using a separate network to estimate the target Q-values, i.e., the DQN uses two neural networks with different parameters but the same structure, as shown in Figure 4. This helps to stabilize the learning process by reducing the variance in the target values used to update the Q-network.

#### 3.2.1. MDQN Training Phase

The MDQN training process in this paper involves the interaction between the agent and the environment simulator, which generates training data. A double network structure is employed, consisting of a Q-network and a target Q-network with identical initial parameters. The agent generates experience by taking actions in the environment and storing the resulting state, action, reward, and next state in a memory pool. To train the neural network, a small batch of data is randomly selected from the experience pool, and a small batch gradient descent method is used to optimize the loss function. This method is less volatile than the random gradient method, reduces variance, and ensures stability. Additionally, small batch training has faster learning speeds and consumes less memory. In this paper, small batches of 50 data points are extracted for training.

The loss function is defined as the deviation of the target network from the current output as follows, which is optimized during training.

(15) $$L\left(\theta \right)=E\left[{\left(Q_{\mathrm{target}\;}-Q\left(s,a,\theta \right)\right)}^{2}\right]$$

(16) $$Q_{target}=r+\gamma max_{a}Q\left(s^{\prime },a^{\prime },\theta \right)$$

The DQN algorithm uses a target Q-value network that is separate from the Q-value network. The target network is kept fixed for a certain number of iterations, while the primary Q-value network is updated using backpropagation. Every *C* iterations, the weights of the primary network are then copied over to the target network, which is used to compute the Q-value targets for the next batch of experiences. This delayed update strategy helps to reduce the risk of oscillations and divergence in the learning process.

The specific training phase is shown in Algorithm 1.
**Algorithm 1:** MDQN training phase for V2V resource allocation1:**Input:** V2V link settings $B_{k}=B$ and $T_{k}=T$ for all k∈K2:**Output:** Trained *Q*-value function Q(s,a,θ) and target *Q*-value network3:Activate environmental simulator and generate vehicles4:**For** each V2V link k∈K:5:     **For** each step:6:          Select subchannels and transmit power based on policy7:          Receive feedback on status and rewards of actions from ambient simulators8:          Collect and store data quadruplet state, reward, action, previous state in memory bank9:          Select a small batch of data from experience pool to train neural network10:        Perform gradient descent according to Equation (20)11:        If step is a multiple of *C*:12:               Copy *Q*-value network weights to target *Q*-value network13:      **End For**14:**End For**15:**Return:** Trained *Q*-value function Q(s,a,θ) and target *Q*-value network

#### 3.2.2. MDQN Testing Phase

In the testing phase, the trained neural network model is loaded. Then, the algorithm’s performance is evaluated by changing the load or noise power of the V2V link in an environmental simulator, which generates vehicles and V2V links.

Each V2V link is selected as an agent, and the action with the largest Q-value is chosen by the agent. The environment is updated according to the chosen action, which results in a change in the state information provided by the environment simulator. The simulator returns a reward value to the agent, and the evaluation results, including the total transmission rate of V2V and the V2V link load transmission success rate, are provided by the environment simulator.

Since the agents select actions independently based on local information, simultaneous action updates can cause the agents to be unaware of the actions taken by other V2V links. This means that the state observed by each V2V link may not fully capture the environment. To address this issue, the agents update their actions asynchronously, meaning that only one or a small fraction of the V2V agents update their actions at each time period. This allows the changes in the environment caused by the actions of other agents to be observed. The specific MDQN testing phase is shown in Algorithm 2.
**Algorithm 2:** MDQN testing phase for V2V resource allocation1: Load the trained network model.2:Activate the environmental simulator and generate vehicles.3:Iteratively select V2V links in the system.4:**for** each V2V link k∈K:5:     select the action with the largest value.6:Update the environment simulator based on the selected action.7:Update the evaluation results, including the total transmission rate of V2V and the V2V link load transmission success rate.8:Return the evaluation results.

## 4. Simulation Analysis

### 4.1. Simulation Parameter Setting

In this paper, the environment simulator is established based on the environment simulation approach proposed in the literature [24], employing the city case evaluation method of the Manhattan model. The simulated environment has dimensions of 1299 m (length) and 750 m (width). A total of *M* vehicles and the base station constitute *M* V2I links. Additionally, each vehicle can establish communication with one neighboring vehicle, resulting in *M* V2V links. These V2V links share *M* subchannels, each with a bandwidth of 0.9 MHz. To model the vehicle load and time delay, the TR 37.885 [26] low-density communication model is employed, specifically the traffic model presented in Section 6.1.5 of TR 37.885 [26]. Table 1 provides an overview of the key simulation parameters utilized in the study.

The link channel model includes path loss, shadow fading, and fast fading. TR 38.901 [27] 7.4.1-1 for V2I links uses the town macrocellular model, considering only the line-of-sight (LOS) case; the V2V link uses the TR 37.885 6.2.1-1 model for path fading, including LOS and non-line-of-sight (NLOS) cases. The model’s large-scale fading is updated at each round, i.e., every 100 ms, while the small-scale fading is updated at each step, i.e., every 1 ms. The specific parameters are shown in Table 2.

### 4.2. Analysis of Simulation Results

**Figure 5:**
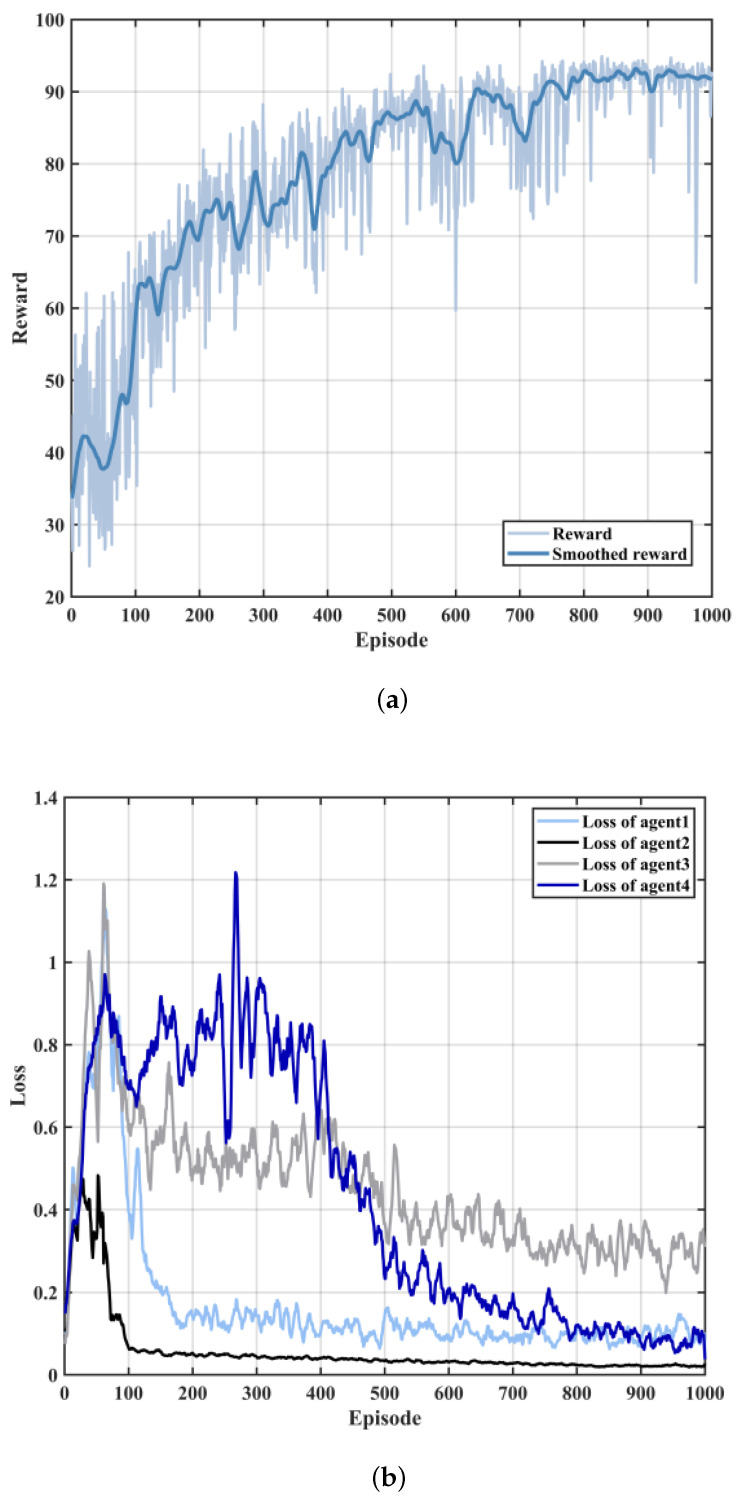
Relationship between reward and loss functions and number of iterations in MDQN algorithm. (**a**) Relationship between reward and number of iterations. (**b**) Loss function versus number of iterations.

Figure 5 shows the average reward and loss values per round as the number of training iterations increases, to show the convergence of the MDQN algorithm. It can be seen from the figure that the cumulative discounted reward per round is increasing and the loss value is decreasing as the training proceeds, which demonstrates the effectiveness of the proposed training algorithm. When the number of training rounds is around 800, despite some fluctuations due to dynamic channel fading in the vehicular network environment, convergence is reached overall with a cumulative discount return of around 92 and a loss value reduced to the order of 0.01.

The proposed 5G slice-based deep learning model is compared with the following three resource allocation schemes.

1. A random resource allocation scheme that randomly selects the spectrum subchannels and transmission power for each V2V link at each time step.
2. A single-agent DQN (SDQN)-based algorithm [28], where, at each moment, only one agent updates its actions based on local observations, while the sub-bands and power of the other agents remain unchanged and all agents are trained together and use the same DQN.
3. The Deep Deterministic Policy Gradient (DDPG) algorithm [29], where the resource allocation problem is solved via the DDPG method.

**Figure 6:**
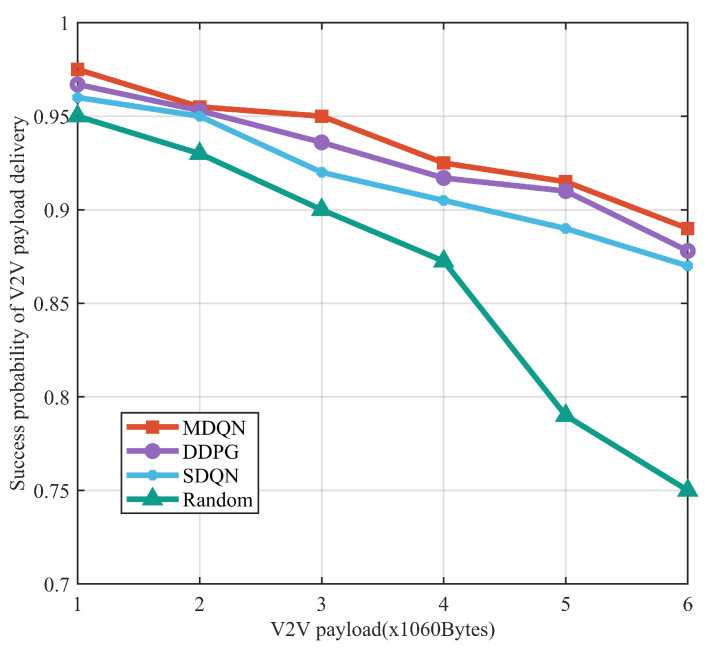
Load transmission success rate versus V2V link load.

It can be seen in Figure 6 that the transmission success probability of all algorithms decreases as the load on the V2V link increases, but the curve of the MDQN algorithm proposed in this paper decreases more gently, indicating that the algorithm can better slow down the performance degradation for high load communication demands. The success rate of the MDQN algorithm is greater than that of the other three algorithms for all load sizes tested. The success rate of the MDQN algorithm is greater than 95% under small loads, and the transmission success rate of this algorithm is also greater than 89% under large loads, while the DDPG algorithm, SDQN algorithm, and random assignment algorithm yield values of only 88%, 87%, and 75%.

**Figure 7:**
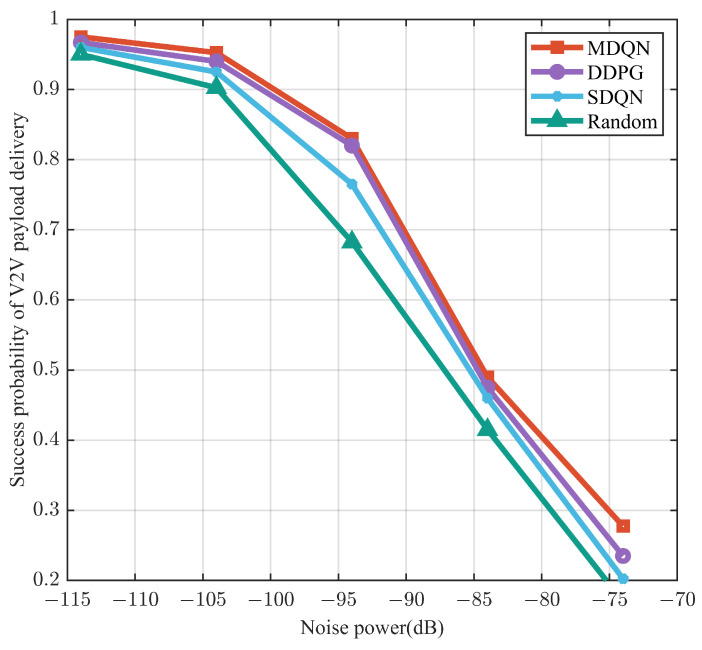
Load transmission success versus noise power.

It can be seen from Figure 7 that the transmission success probability of all algorithms decreases as the noise power increases, and the transmission success rate probability of the other three resource allocation methods is significantly lower than that of the MDQN algorithm. In addition, even though the MDQN algorithm is trained with a fixed noise power of −114 dB, it achieves a higher load transfer success probability than the other three benchmark schemes at different noise powers, reflecting its robustness to noise variations.

Table 3 shows the SEE of the different resource allocation schemes under different V2V loads. It can be seen that the SEE of the proposed MDQN method is greater than that of the other three schemes.

**Figure 8:**
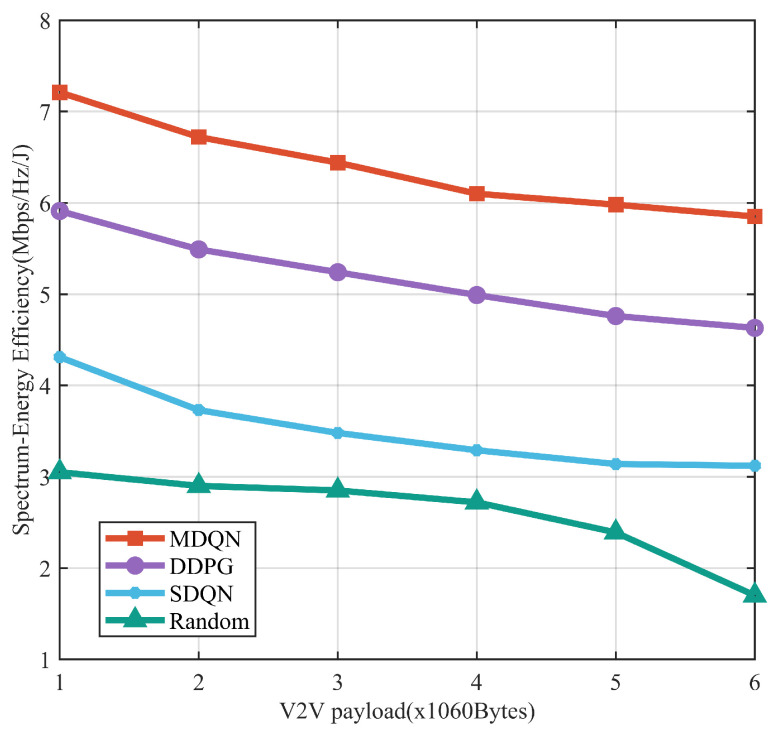
Network SEE versus V2V link load.

The performance variation of the network SEE when the V2V link load varies is shown in Figure 8. As the load increases, the V2V link is more likely to choose a large transmit power in order to improve the transmission success rate and the transmission time will be prolonged, resulting in an increase in the intensity and time of interference with other links, so the performance of all schemes degrades. However, the network SEE of the MDQN algorithm proposed in this paper is always higher than that of the other three resource allocation schemes.

**Figure 9:**
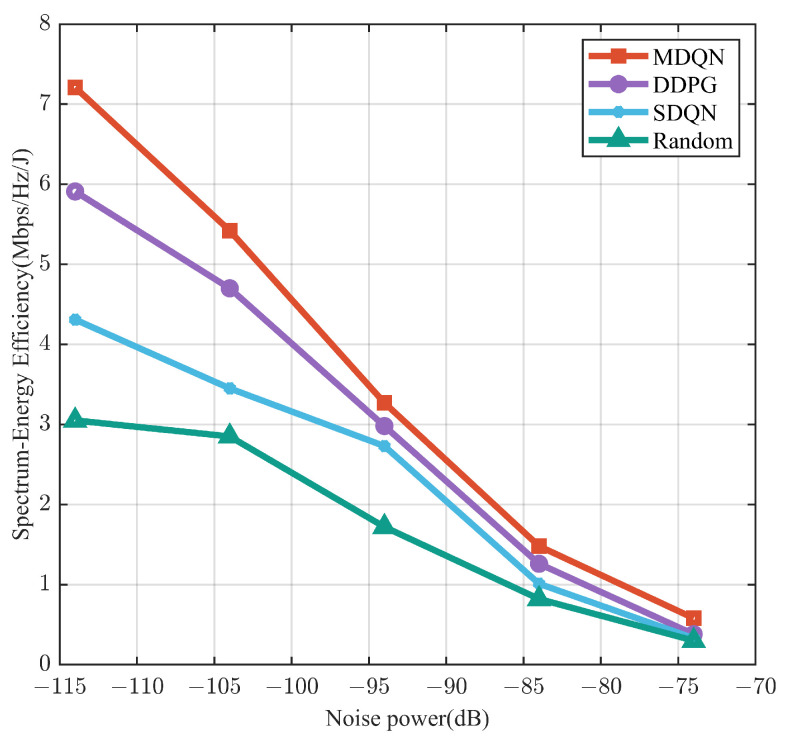
Network SEE versus noise power.

The performance of the network SEE of the algorithms for different noise powers is shown in Figure 9. As the noise power increases, the network SEE of all algorithms decreases, but the network SEE of the proposed deep learning is always larger than that of the other two algorithms. The SEE of the MDQN algorithm is 7.21 Mbps/Hz/J under the noise power −114 dB, while the DDPG algorithm, the SDQN algorithm, and the random assignment algorithm yield values of only 5.91 Mbps/Hz/J, 4.31 Mbps/Hz/J, and 3.05 Mbps/Hz/J.

## 5. Conclusions

This paper mainly investigates the wireless resources of 5G networks based on V2V communication under 5G NR V2X standard and network slicing technology. V2V links and V2I links use hybrid spectrum access, with maximizing the network SEE as the optimization objective, incorporating reliable transmission and delay constraints of URLLC slicing into the optimization problem and using a deep reinforcement learning approach with multiple agents to seek a V2V communication resource allocation strategy. The simulation results demonstrate that the proposed MDQN algorithm significantly enhances the network SEE while maintaining a high success rate for V2V link transmissions. Specifically, when the noise power is set at −114 dB, the MDQN algorithm achieves SEE of 7.21 Mbps/Hz/J and a transmission success probability of 0.98. In contrast, the SEE values for the comparative algorithms, namely the DDPG algorithm, the SDQN algorithm, and the random allocation algorithm, are only 5.91 Mbps/Hz/J, 4.31 Mbps/Hz/J, and 3.05 Mbps/Hz/J, respectively. Compared with the three mentioned comparative algorithms, the MDQN learning training model studied in this paper enables V2V links to learn to select transmit power and subchannels to achieve better performance when the environment changes. Furthermore, the extension of the MDQN algorithm to address the joint computation offload and resource allocation problem represents a promising avenue for further investigation in future research.

## Figures and Tables

**Table 1:** Simulation parameters.

Parameters	Value
Number of subchannels *M*	4
Number of V2V links	4
Carrier frequency	4.7 GHz
Bandwidth	0.9 MHz
Noise power of vehicle receivers	9dB
Vehicle movement speed *v*	36 km/h
V2V transmission power	[23,15,10,5,−100] dBm
Noise power	−114 dBm
Circuit power	16 dBm
V2V link load transmission tolerance delay *T*	100 ms
V2V Load *B*	[1, 2, …] × 1060 Bytes
Signal-to-noise ratio threshold	1 dB

**Table 2:** Channel model.

Parameter	V2I Link	V2V Link
Path loss	$28+22\log_{10}\left(d\right)+20\log_{10}\left(f_{c}\right)$	LOS: $38.77+16.7\log_{10}\left(d\right)+18.2\log_{10}\left(f_{c}\right)$ NLOS: $36.85+30\log_{10}\left(d\right)+18.9\log_{10}\left(f_{c}\right)$
LOS probability	1	VUE pairs on the same path
Shadow decay distribution	Log-normal distribution	Log-normal distribution
Standard deviation of shadow decay	LOS: 4 dB NLOS: 6 dB	LOS: 3 dB NLOS: 3 dB
Go to the relevant distance	50 m	10 m

**Table 3:** SEE (Mbps/Hz/J) of different resource allocation schemes under different V2V loads.

	Scheme	MDQN	DDPG	SDQN	Random
V2V Load					
ine 1060 Bytes		7.21	5.91	4.31	3.05
2120 Bytes		6.72	5.49	3.73	2.90

## Data Availability

Not applicable.
